# Experimental Investigation of the Effect of Surfactant–Polymer Flooding on Enhanced Oil Recovery for Medium Crude Oil

**DOI:** 10.3390/polym16121674

**Published:** 2024-06-12

**Authors:** Oluwasanmi Olabode, Humphrey Dike, Damilola Olaniyan, Babalola Oni, Michael Faleye

**Affiliations:** Department of Petroleum Engineering, Covenant University, Ota 112104, Ogun State, Nigeria; humphrey.dike@covenantuniversity.edu.ng (H.D.); damilola.olaniyan@covenantuniversity.edu.ng (D.O.); oni.babalola@und.edu (B.O.);

**Keywords:** polymer, surfactant, enhanced oil recovery, core flooding, polymeric surfactant

## Abstract

High technical and financial risks are involved in exploring and exploiting new fields; hence, greater focus has placed on the development of environmentally friendly, cost-effective, and enhanced oil recovery (EOR) options for existing fields. For reservoirs producing high-density crudes and those with high interfacial tensions, water flooding is usually less effective due to density differences—hence the advent of polymer and surfactant flooding. For cost-effective and eco-friendly EOR solutions, a biopolymer and a surfactant synthesized from Jatropha seeds are used in this study to determine their effectiveness in increasing the oil recovery during core flooding analysis. The experiment involved an initial water flooding that served as the base cases of three weight percentages of polymers and polymeric surfactant solutions. The results for the polymer flooding of 1 wt%, 1.5 wt%, and 2 wt% showed an incremental oil recovery in comparison to water flooding of 16.8%, 17%, and 26%, while the polymeric surfactant mixtures of 5 wt% of surfactant and 1 wt%, 1.5 wt%, and 2 wt% of a polymer recorded 16.5%, 22.3%, and 28.8%, and 10 wt% of surfactant and 1 wt%, 1.5 wt%, and 2 wt% of a polymer recorded incremental oil recoveries of 20%, 32.9%, and 38.8%, respectively.

## 1. Introduction

Industrial expansion and rising populations have made the demand for energy rise exponentially. Due to the depletion of most reservoirs around the world and the financial and technical risks involved in venturing into the exploration and exploitation of new reserves, various techniques have been initiated to further deplete hydrocarbon reserves from existing reservoirs. Secondary recovery schemes are usually initiated at the onset of the initial production from natural drives (if required) or when natural drives have been depleted. In either case, recoveries from secondary recovery schemes are usually low, especially for medium to heavy crude reservoirs. Enhanced oil recovery (EOR) options for such reservoirs are normally screened and ranked based on the costs, environmental impact, and oil recovery factors [1,2]. 

Thermally enhanced oil recovery options when implemented in medium-heavy crude reservoirs reduce the oil viscosity but comes with challenges of a loss in the lighter components of hydrocarbons, reduction in reservoir permeability due to coke formation (for in situ combustion), and heat loss in highly fractured reservoirs [3]. Certain issues are often associated with availability and cost-effectiveness for gas injection or steam injection; thus, chemical options of enhanced oil recovery are mostly preferred. However, [2] pointed out the highs and lows of most chemical EORs. Even though they are successful in enhancing oil recovery, they are not environmentally friendly and mostly costly, and the oil recovery is sometimes low. 

Chemical EOR strategies vary with respect to the prevailing reservoir fluids and rock properties and involve various mechanisms such as reducing interfacial tension [4], altering surface wettability [5], mobility control by using high viscosity agents such as the polymer [6] application of thermal processes to reduce the viscosity of oil by increasing the reservoir temperature through processes such as in situ combustion, steam flooding, and hot water injection [7], and the application of microbes for oil recovery for depleted reservoirs [8]. EOR processes can include one or more of these mechanisms and to be successful, the methods must be profitable, practical, and reliable [9].

In their studies, ref. [10] deduced that increasing the surfactant concentration in a reservoir system of heavy crude/brine/surfactant reduces the interfacial tensions of the fluid contact system. This method has been singularly used to increase the oil recovery in oil rim reservoirs and in conjunction with gas injection to create a foam barrier against an expanding gas cap [11]. Reducing the saline concentrations of injected water (an option for low-salinity water flooding) has been studied to increase the oil recovery in medium to heavy crude reservoirs [12]. The factors such as high concentrations of brine and temperatures are amongst the main reservoir uncertainties that affect the effectiveness of surfactant flooding [13].

**Figure 1:**
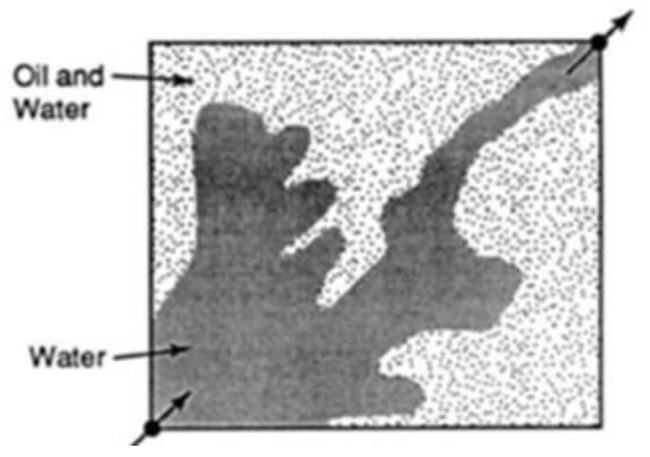
Irregular pattern under water flooding. Source: [15].

**Figure 2:**
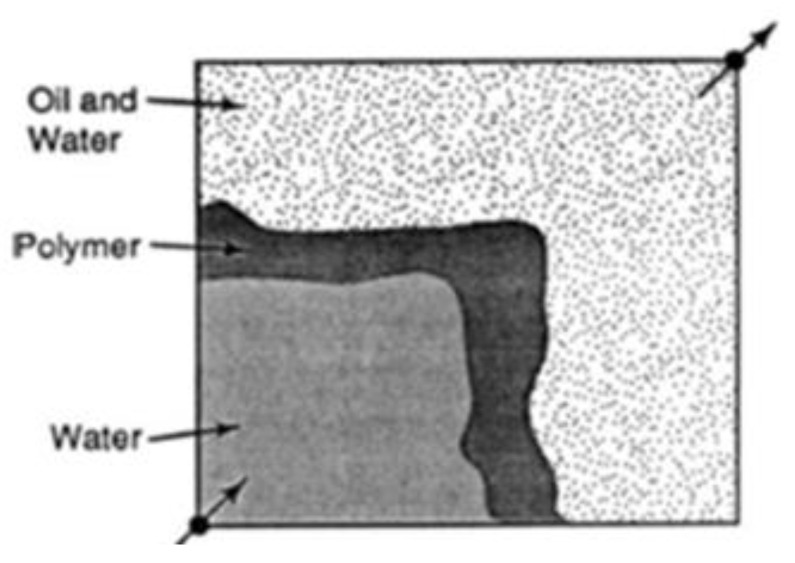
Regular polymer flood pattern. Source: [15].

During water flooding, injected water flows quicker than the crude oil (medium/heavy crude) in the reservoir because of its low viscosity. As a result, an irregular oil front is formed that leads to water penetrating the petroleum front, leaving regions with unswept petroleum [14]. This is otherwise known as viscous fingering of the injected fluids through the region of high-viscosity fluids. The addition of polymers to the injected water increases the viscosity of the water and reduces the permeability to water due to the mechanical entrapment of the polymer, thus decreasing its mobility and creating a uniform sweep from the injector to producer wells. Figure 1 and Figure 2 show the irregular and regular flood patterns encountered during water and polymer flooding, respectively. A substantial volume of oil is left behind in Figure 1 compared to Figure 2 that has a near uniform flood flow through the reservoir. The arrows at the bottom left and upper right are describing the locations of the injector and producer wells respectively. 

Due to the different types of polymers (synthetic and biopolymers), polymers work in different conditions, thus offering differing oil recovery factors [16]. During the screening criteria for EORs (especially under polymer flooding), reservoir factors such as permeability, IFTs, the viscosity of oil, wettability, temperature, salt concentrations, and polymer retention or adsorption on rock surfaces are essential to be considered to determine the successful implementation of polymer injection [16]. The inherent/in situ state of these variables have led to the use of different EOR combinations which is slowly becoming a phenomenon for oil recovery compared with singular deployment that often leads to low oil recovery. Various combinations of different EOR methods such as Polymer-coated Nanoparticles (PNP) and Polymer Surfactants (PS) have been studied with core flooding experimental applications by [17,18]. The summary of their results shows that the PNPs can alter the rock wettability, hence increasing the oil recovery factors. The use of nanoparticles has been explored in wider and remarkable areas of application such as polymer composites for enhanced oil recovery [17] and drug delivery [19]. Simulation studies on polymer flooding studied by [20] confirms that the sequential injection of different polymer concentrations will improve the area sweep efficiency for heterogeneous reservoirs. The observations from simulation studies were the failure to indicate the critical polymer concentration that will yield the maximum oil recovery, the effect of reservoir temperature, and polymer adsorption indexes. Simulation studies on the effects of salt concentrations on polymer flooding were carried out by [21] and it is significant to note that higher salt concentrations in the reservoir/injecting fluids will have an adverse effect on the oil recovery. Surfactants achieve success as EOR processes must transcend interfacial properties as they must have the ability to migrate through porous media and be dispersible in water/brine solutions, and be of low cost and injectable into the reservoir. Being interfacially active is a major challenge as the applications of PNP can lead to the plugging of pore spaces (especially at high nanoparticle concentrations) [22], the swelling of clay [23], stability, and adsorption on rock surfaces [9].

The application of polymers and surfactants as a chemical-enhanced oil recovery (EOR) technique has become a promising technique for the efficient recovery of oil [5,24,25]. Biosurfactants and biopolymers are of importance as most are derived from non-edible seeds which are inexpensive, readily available, and environmentally friendly [26]. Various methods have been employed to extract surfactants from solid and liquid components [27]. From the studies of [28], anionic surfactants are synthesized from soapnut oil through a trans-esterification process using fatty acids followed by sulfonation of the fatty acid methyl ester. An experimental sensitivity analysis of the factors that affect surfactant solutions during enhanced oil recovery processes was conducted by [27]. Their results indicated an incremental oil recovery of 13% via surfactant slug injection. The phase behavior of microemulsions stabilized with surfactants derived from Jatropha oil was studied by [29]. They estimated an average of 31% incremental oil recovery during the slug injection of surfactants and concluded that microemulsions stabilized with surfactants have effective interfacial, rheological, and flooding properties to recover residual oil. It is important to note that the addition of surfactants to an emulsion mixture (emulsification process), such as polymers, helps to effectively increase the viscosity of the displacing fluids and drastically reduce the interfacial tension. The effect of the emulsification on the performance of enhanced oil recovery is the control of the mobility of the displacing fluid and increase in the swept volume. 

In many applications, Jatropha oil, a non-edible oil, has received considerable attention for its extraordinary properties, particularly in the detergent industry. Jatropha Curcas grows in low-fertility soils and humid conditions and can withstand very high temperatures. Previous research such as [30,31,32,33] has acknowledged the use of Jatropha oil and the manufacture of biodiesel. Jatropha oil seeds are a good source of lipid, and its oil content is approximately 20 to 40%, which provides a good justification for its use as a source of soap for energy and the detergent industry. While describing the methods for extracting, synthesizing, and characterizing castor oil seeds, Ref. [34] concluded that the biosurfactants thus derived from an alkaline base normally contain fatty acids with a content of about 87%. The direct reaction of pyrolysis was used to extract a surfactant from castor seeds as part of it is used for enhanced oil recovery [34]. The resultant mixture greatly reduced the interfacial tensions between the fluid surfaces resulting in oil recoveries of up to 60%. After the extraction of a surfactant from Jatropha seeds, Ref. [35] measured the interfacial force responses, viscosities, surface tensions, and contact angles of different concentrations of the polymeric surfactants (PSs) used for enhanced oil recovery. Their studies concluded that increasing the PS concentration to 8 g/L could further increase the oil recovery to 28%. An outline of the various advantages of the use of PS injection was given by [36] and included increasing the viscosity of the displacing fluid, a reduction in the mobility ratio, a reduction in the interfacial tension between oil and water, the formation of interfacial films that prevent oil surfaces from coalescing thereby aiding the flow of the oil phase, and the enhancement of oil recovery. Their study included water in oil emulsion flooding which to some extent improves the oil recovery but increases the overall cost of de-emulsifying the produced oil.

Chemical-enhanced oil recovery options are quite expensive and to obtain maximum recovery, two or more chemical options are usually combined after ranking the various options based on the reservoir and fluid properties. Most EOR options are also deployed after the secondary techniques have been exhausted; thus, the reserves recoverable under EOR schemes are usually of lower volumes compared to primary and secondary recovery. Thus, there should be a cost justification for implementing an EOR technique with respect to its recoverable reserves and their effect on the environment. Although the process of synthesizing Jatropha seeds to extract the oil (especially in large quantities) might be costly, the seeds are non-edible and less expensive. Guar gum is a readily available polymer with enhanced rheological properties and is less expensive. A PS solution option in EOR offers a wider range of other advantages such as a reduction in the interfacial tensions between oil and water, wettability alterations, and increasing the viscosity of the injection fluid [37]. Using a polymer (partially hydrolyzed polyacrylamide) and surfactant (sodium dodecyl sulfate) [38] recorded an incremental oil recovery of 23% and 20% during surfactant flooding as opposed to water flooding. From the PS experimental flooding carried out by [39], the rapid emulsification of oil led to a substantial increase in the oil recovered compared to water and polymer flooding. A synthesis of Fatty Acid Methyl Ester and Ethyl Acrylate by [40] to produce a PS resulted in a 0.5% increase in oil recovery compared to water flooding. Core flooding experiments by [41] described methyl ester sulfonate as a PS to be very thermally stable and increase the oil recovery by up to 10% above that recorded during water flooding. Due to the unique properties and excellent performances of PSs during pilot tests where incremental oil recoveries of about 20% were recorded, they have been considered to replace normal polymer flooding in highly heterogenous reservoirs [42]. PSs synthesized from sodium methyl ester and castor oils reduced the IFT to an ultra-low value, and this mixture resulted in a 29.1% increase in the oil recovery during a sand pack experiment [43]. Core flooding experiments were conducted in a sand pack system, to study the EOR efficiency using the synthesized PS, and more than 26% of additional oil recovery was observed after normal water flooding [36]. The aim of this study was to obtain different polymeric surfactants for core flooding experimentation on the enhanced oil recovery. This study first synthesized surfactants from Jatropha seeds using the sulfonation method and then developed different mixture concentrations using Guar gum with the aim of estimating the oil recoveries (from a core flooding experiment) at those concentrations with a comparison of the results with those obtained from water flooding. 

## 2. Methodology

### 2.1. Experimental Setup and Procedures

**Figure 3:**
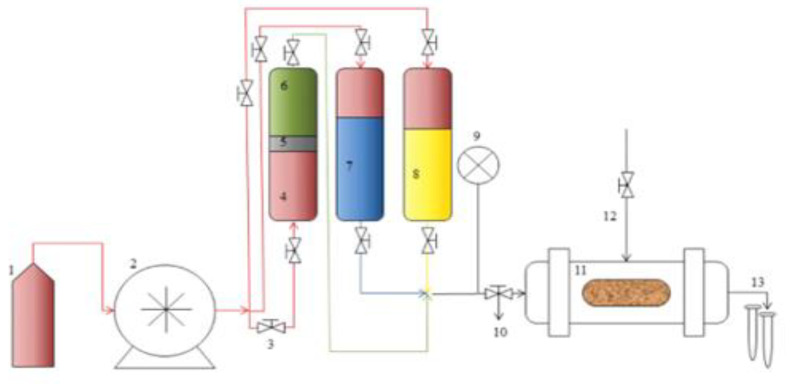
Schematic for the core flooding tester [44].

The experimental study was conducted at the Department of Petroleum engineering, Covenant University. The first procedure was to prepare the core samples using equipment such as the following:

1. Soxhlet Extractor: a single unit extractor from Vinici Technologies, Nanterre, France this used to remove water and oil from core samples and restore the core to its original state or prepare it for flooding purposes. It has the capacity to heat 250 mL of water to about 450 °C.
2. Manual Saturator: The manual core saturator from Vinici Technologies, Nanterre, France is used to obtain remarkable fluid saturations of dry core samples. It has a cell diameter and height of 58 mm and 300 mm respectively.
3. Pycnometer: this is used to determine the original densities of the crude oil sample and polymeric surfactant mixtures.
4. Glass capillary viscometer: The OFITE viscometer, (Product Code: 130-10-L-99, Houston, TX 77065 USA) is used to measure the viscosity of fluid samples.
5. Desiccator: its sole purpose is to dry the core samples in a vacuum. The system includes a heated desiccator and a vacuum pump.
6. Reservoir Permeability Tester: The OFITE Permeability tester (Product Code: 127-00, Houston, TX 77065 USA) has various functions such as testing core samples and measuring their permeabilities, and core flooding analysis such as water flooding, gas injection, and enhanced oil recovery options. These enable the parameters like water and oil saturations, oil recoveries, and residual saturations to be measured. The schematic for the equipment is shown in Figure 3 and can be configured to suit various functions [6].

The equipment consists of 3 pressure systems, namely:

1. Confining pressure (to maintain the pressure of the core and helps to avoid the displacing fluids from going outside the core).
2. Drive pressure (indicated by 9 which controls water pumps and hydraulic fluids used in pushing fluids into the core).
3. Back pressure which is set to keep the displacing fluids from boiling at the test temperatures.

Different fluid samples to be used are filled in 1, the fluid pump indicated by 2 is used to fill or re-fill the accumulators (4, 5, 6, 7, and 8) with the fluids to be injected. The valves indicated by 3 are used to isolate a fluid accumulator when another fluid is to be injected. The core samples are placed in the core holder labeled 11, and the effluents of oil and water are collected in test tubes indicated by 13. The tests were carried out at a differential pressure of 27 psia and temperature of 27 °C.

The materials used included crude oil (API 22°) from the Niger Delta oil-producing region of Nigeria, Jatropha seeds from a local market in Lagos, Nigeria, Guar gum (95% pure), deionized water, toluene, and chemical reagents such as standard sodium carbonate (0.5 M), hydrochloric acid (0.5 M), alcoholic potassium hydroxide (0.5 M solution in 96% ethanol), phenolphthalein indicator (1% solution in 96% ethanol), and sodium hydroxide, which were all obtained from Abams Chemicals in Lagos, Nigeria.

### 2.2. Sulfonation Method

**Figure 4:**
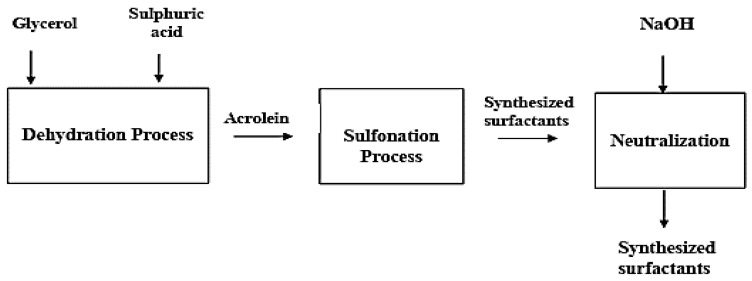
Flow chart of surfactant production via sulfonation/formulation.

The schematic for the sulfonating process is shown in Figure 4. A beaker with a motor stirrer was used to combine vegetable oil, sulfuric acid, and glycerol. The glycerol in the presence of a catalyst (sulfuric acid (H_2_SO_4_)) was formed via the dehydration of Acrolein. In the development of detergents and acrylic acid esters, acrolein is an essential and versatile chemical intermediate.

In the formulation cycle, a glass device fitted with a motorized blowing agent at a temperature of 40–45 °C was connected to a chamber containing a 95% concentration of glycerol sulfuric acid for over two hours. By pouring the mixture over glass, the sulfonated mass was cooled to 20 °C and was neutralized with 50 wt% of caustic soda (NaOH). Through this process, the surfactant concentration was confirmed to be soluble in water.

The indication of the presence of murky soapy water (soap cloudiness) signifies micelles’ development during the surfactant synthesis [4,45]. They concluded that the murkier the water becomes, the more micelles that are created via the detergent’s washing capabilities. The polymer used in the study was weighed out in different amounts in grams using an electric weighing balance; the respective weights are shown in Table 1. Each of these weights were then dissolved in a base fluid (water) of 500 mL. A centrifuge meter was utilized to thoroughly combine the mixture to form a single phase. The next phase was to separately weigh 5 wt% and 10 wt% of the surfactant and mix it with the resulting compositions above to obtain various polymeric surfactant compositions. This gave a total of 10 polymeric surfactant concentrations. These concentrations were subsequently used for the flooding process.

### 2.3. Characterization of the Core Samples

Normally, a core sample from the representative reservoir in question is needed but in the absence of this, core samples were obtained from producing wells in the Niger Delta region of Nigeria. Core flooding test analysis was conducted on 3 samples to estimate their petrophysical properties as shown in Table 2. The permeability was measured under the influence of differential pressure measured using the core tester. These core samples were used to research the impact of the polymeric surfactant application formulated from Guar gum and Jatropha oil surfactants for the enhanced oil recovery. Equations (1)–(3) were used for estimating the porosity, pore, and bulk volumes of the core samples.

(1) $$V_{b}=\frac{{\pi D}^{2}}{4}$$

(2) $$V_{p}=\frac{saturated\;weight-dry\;weight}{density\;of\;saturant}$$

(3) $$\emptyset =\frac{V_{p}}{V_{b}}$$

where D is the diameter (cm), ∅ is porosity, and V_p_ and V_b_ are pore and bulk volumes, respectively. The underlying mathematical equations and mensuration analysis were used to estimate the oil and water saturations for the core samples.

(4) $$S_{o}=\frac{Total\;volume\;of\;oil\;recorded}{Initial\;Oil\;in\;place}\;or\;\frac{Total\;volume\;of\;oil\;in\;place}{Total\;pore\;volume}$$

(5) $$S_{w}=1-S_{o}$$

(6) $$S_{or}=\frac{Residual\;volume\;of\;water}{Total\;volume\;of\;oil\;in\;place}$$

where S_w_ is the water saturation, S_wi_ is the initial water saturation, and S_or_ is the residual oil saturation.

### 2.4. Characterization of Jatropha Oil

The physiochemical properties of seed oil are shown in Table 3, while the fatty acid content of the seed oil and its ratios are shown in Table 4. Jatropha seed oil has a very high content of palmitic acid. The crude oil used in the main flood tests was purchased from a Nigerian indigenous oil company, and the sample was collected from the Niger Delta oil field. The raw oil (dark brownish in color) collected had an API gravity of 24, specific gravity of 0.89 at 55 °F, and kinematic and dynamic viscosities of 6.5 cp and 5.3 cp, respectively.

### 2.5. Rheological Properties of Biopolymers and Biosurfactants

**Figure 5:**
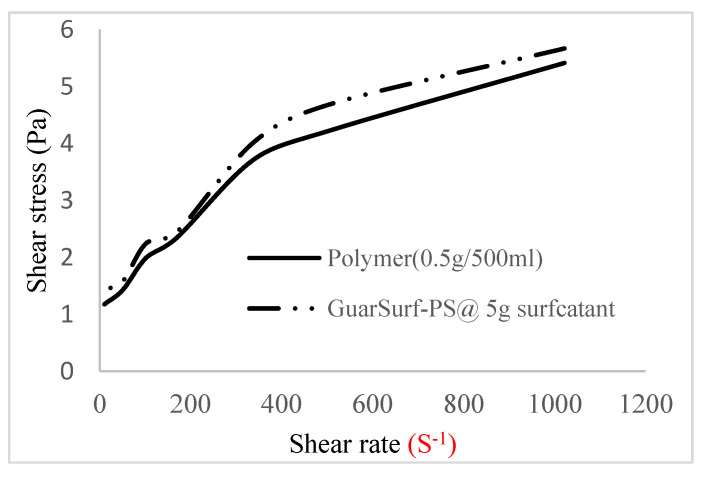
Shear stress vs. rate trend.

**Figure 6:**
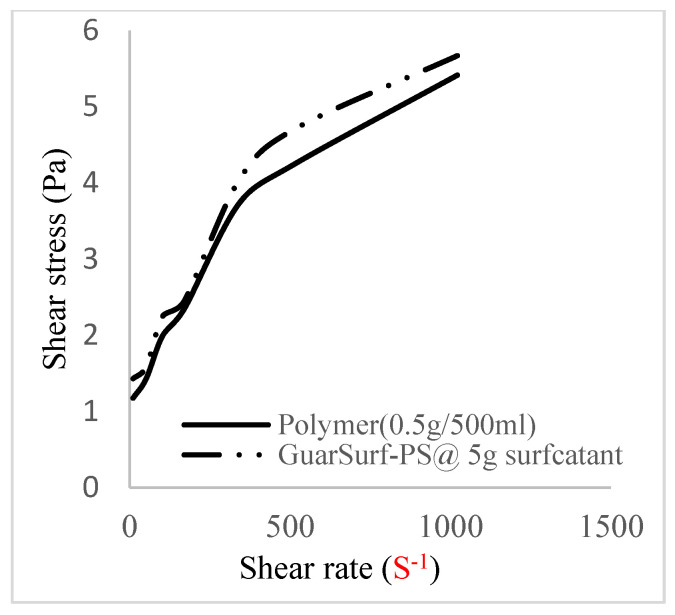
Shear stress vs. rate trend.

**Figure 7:**
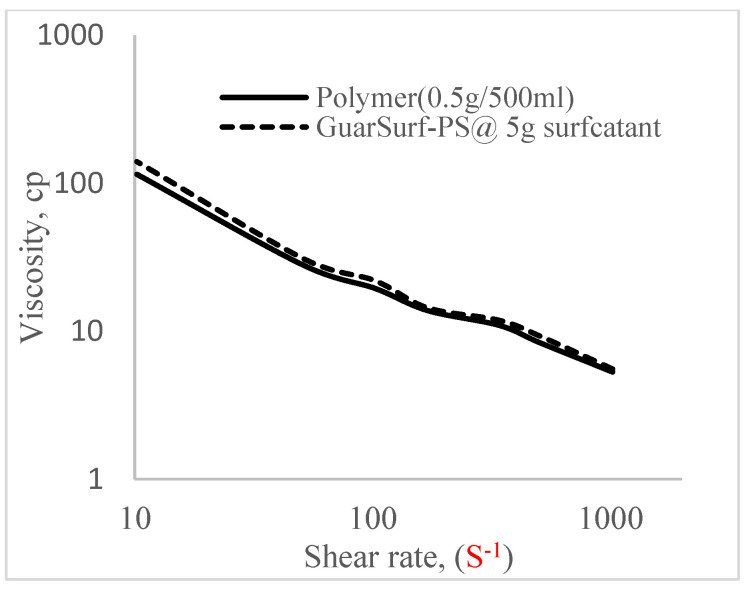
Viscosity trend for polymer @ 0.5 g/500 mL and 5 g of surfactant.

**Figure 8:**
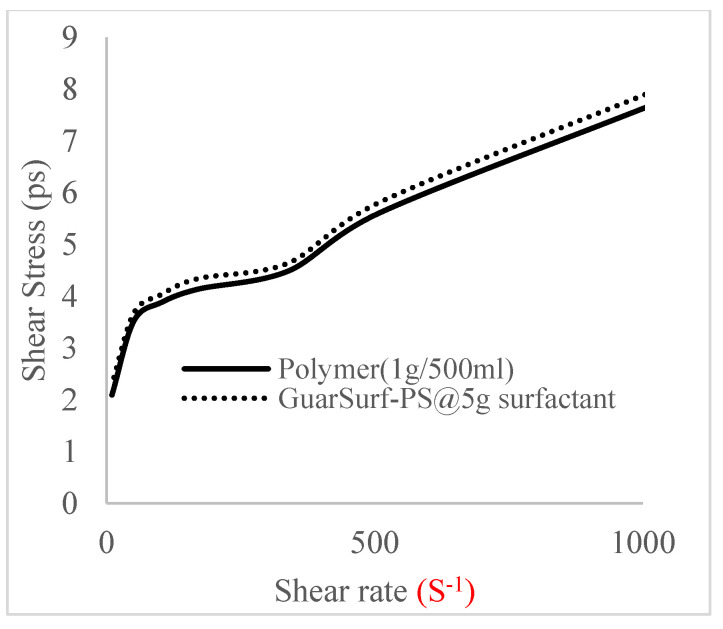
Shear stress vs. rate trend.

**Figure 9:**
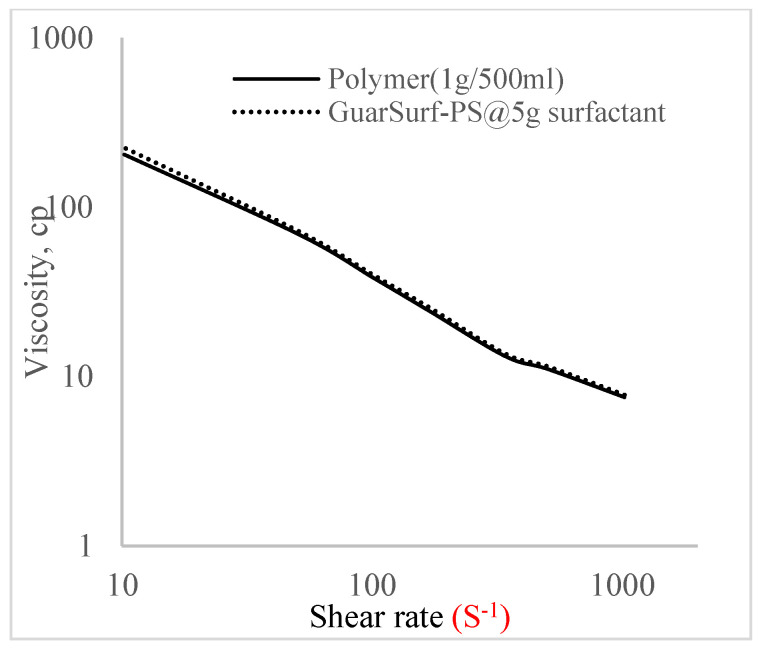
Viscosity trend for polymer @ 1 g/500 mL and 5 g of surfactant.

**Figure 10:**
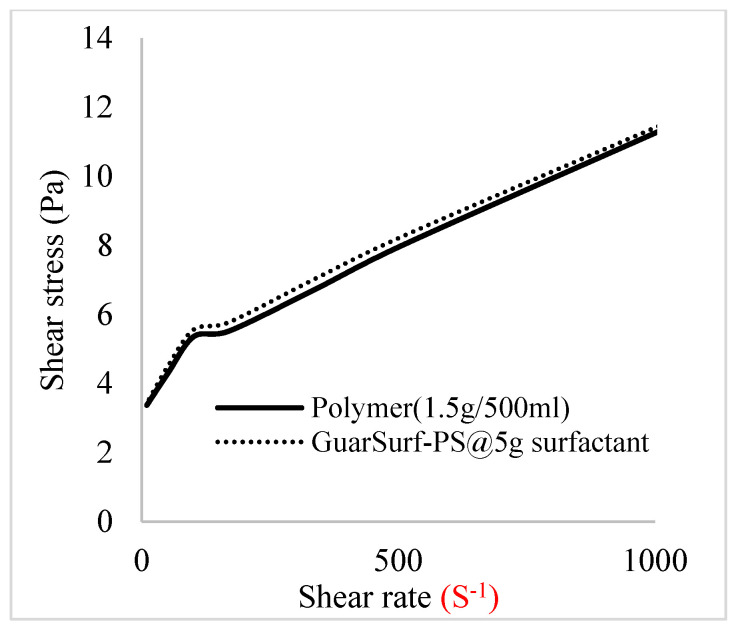
Shear stress vs. rate trend.

**Figure 11:**
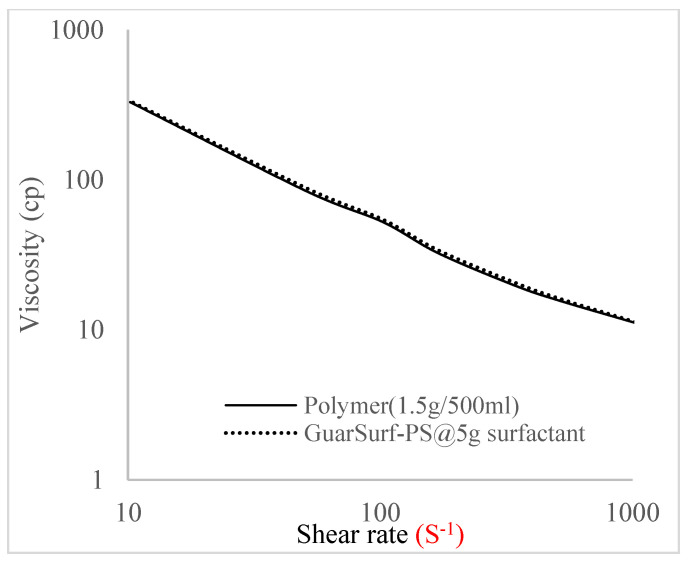
Viscosity trend for polymer @ 1.5 g/500 mL and 5 g of surfactant.

**Figure 12:**
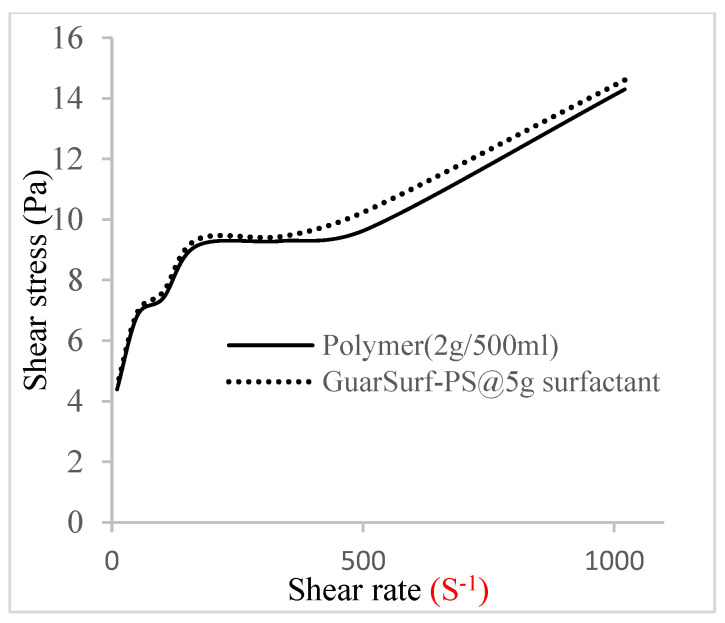
Shear stress vs. rate trend.

Rheological property plots such as the viscosity against the shear rates and the shear stress against the shear rates are important measurements that are necessary to depict the behavior of the mixtures as they undergo stress in a porous media. The relationships between the viscosities, shear rate, and shear stress are described in Figure 5, Figure 6, Figure 7, Figure 8, Figure 9, Figure 10, Figure 11 and Figure 12 below:

1. Rheological behavior of Guar gum @ 0.5 g/500 mL of water and a polymeric surfactant @5 wt% of surfactant mixture (Figure 5 and Figure 6).
2. Rheological behavior of Guar gum @ 1 g/500 mL of water and a polymeric surfactant @5 wt% of surfactant mixture (Figure 7 and Figure 8).
3. Rheological behavior of Guar gum @ 1.5 g/500 mL of water and a polymeric surfactant @5 wt% of surfactant mixture (Figure 9 and Figure 10).
4. Rheological behavior of Guar gum @ 2 g/500 mL of water and a polymeric surfactant @5 wt% of surfactant mixture (Figure 11 and Figure 12).

**Figure 13:**
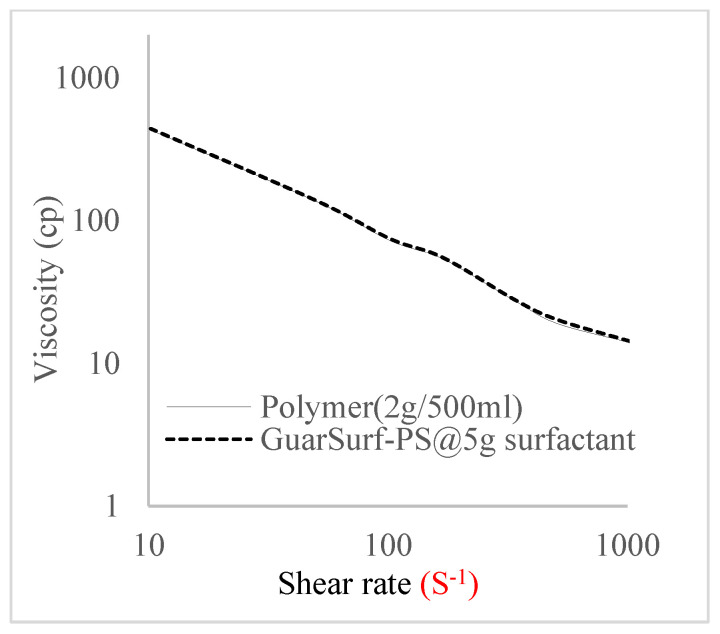
Viscosity trend for polymer @ 2 g/500 mL and 5 g of surfactant.

The shear stress versus shear rate and viscosity versus share rate relationships for the polymer mixtures and polymeric surfactant concentrations are shown in Figure 5, Figure 6, Figure 7, Figure 8, Figure 9, Figure 10, Figure 11, Figure 12 and Figure 13. The charts show an increased viscosity for the polymeric surfactant compared to the polymer concentration.

The last set of rheology tests was performed on different concentrations between the base polymer concentrations (0.5 wt%, 1 wt%, 1.5 wt%, 2 wt%) and 10 wt% Jatropha oil-formulated biosurfactant concentrations. These sets of generated mixtures were allowed to settle for a day; afterwards, the rheology of these mixtures was determined using a viscometer set at different revolutions per minute (rpms), which gave respective dial readings. The rpm readings used were 600 rpm, 300 rpm, 200 rpm, 100 rpm, 60 rpm, 30 rpm, and 6 rpm. A common trend observed for the polymer concentrations was that as the shear rate increased, the viscosity reduced due to increases in shear thinning, and as the shear stress increased, the shear rate increased, which is common for non-Newtonian fluids. It is also expected that the viscosity of the mixture will increase as the weight percentages of the polymer increase. Also, the addition of surfactants to the polymers slightly increased the viscosity of the overall mixture due to an emulsification process as described by [27], and this increases with an increase in the surfactant solution concentration. 

### 2.6. Water Flooding Procedure 

The core samples used for this experiment were already 100% saturated with brine after the cleaning procedure; therefore, it was necessary to introduce oil into the reservoir core plugs. At a fluid flow frequency of 5 mL/min, the crude oil was injected into the core plugs that drained the initial brine in the core sample until no more brine was produced. This process enabled the initial saturation of water, S_wi_, to be established. Following the injection of oil into the core plugs and obtaining a saturation value for the initial water, water was injected at a rate of 3 mL/min. This was achieved in the secondary recovery phase. Water was pumped into the core sample at a steady rate until the core failed to produce any oil. Through this process, the remaining oil saturation of the core plug was determined.

### 2.7. Case Studies

From the description in Figure 5, Figure 6, Figure 7, Figure 8, Figure 9, Figure 10, Figure 11, Figure 12 and Figure 13, 3 polymer concentrations that performed well with respect to viscosity were selected with their resulting mixtures with surfactants. Each of the following case scenarios commenced with initial water flooding. Thus, the case scenarios are shown as follows:

1. Polymer flooding used for core Z (at 1 wt%, 1.5 wt%, and 2 wt% of polymer).
2. Polymeric surfactant flooding used for core C (at 5 wt% of surfactant and 1 wt%, 1.5 wt%, and 2 wt% of polymer).
3. Polymeric surfactant flooding used for K (at 10 wt% of surfactant and 1 wt%, 1.5 wt%, and 2 wt% of polymer).

## 3. Results

### 3.1. Case 1

**Figure 14:**
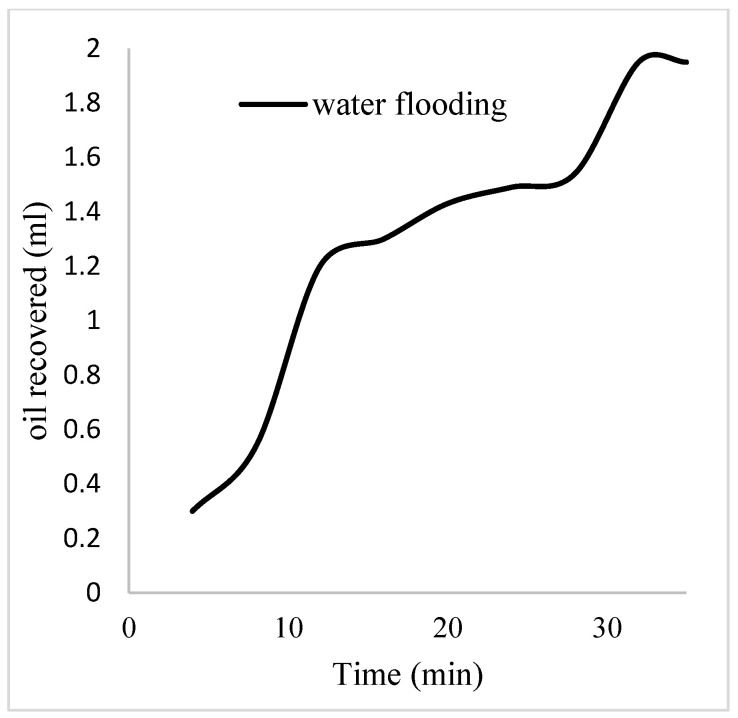
Oil recovery via water flooding.

**Figure 15:**
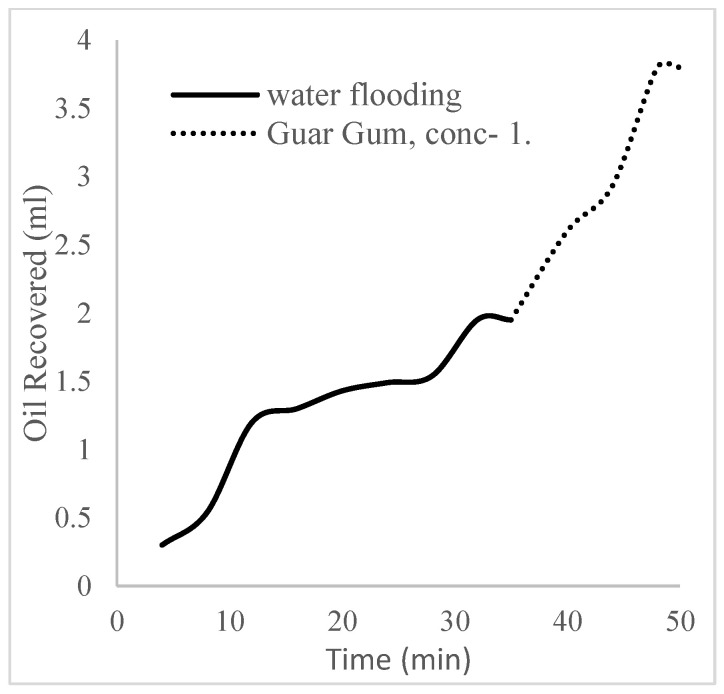
Oil recovery for 0.5 wt% of the polymer.

**Figure 16:**
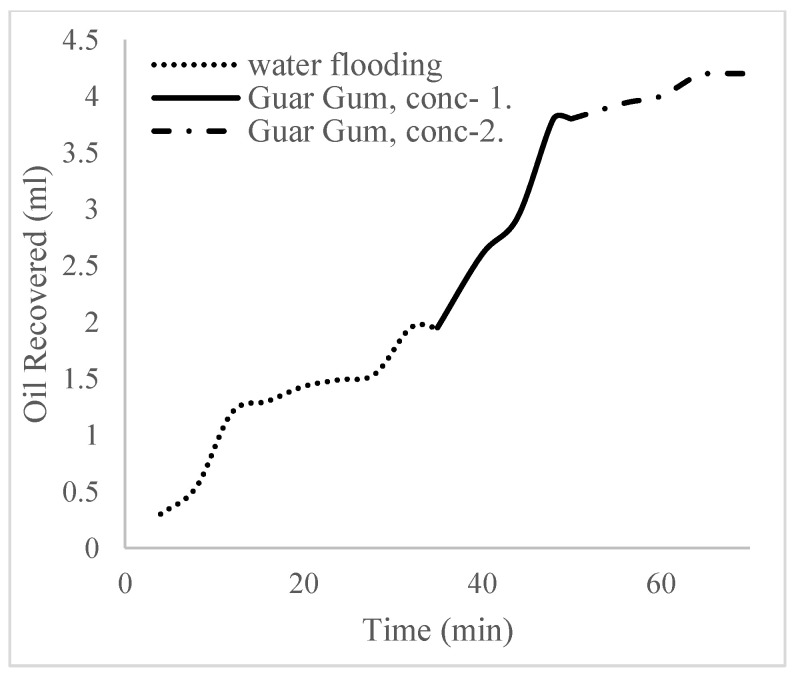
Oil recovery for 1 wt% of the polymer.

**Figure 17:**
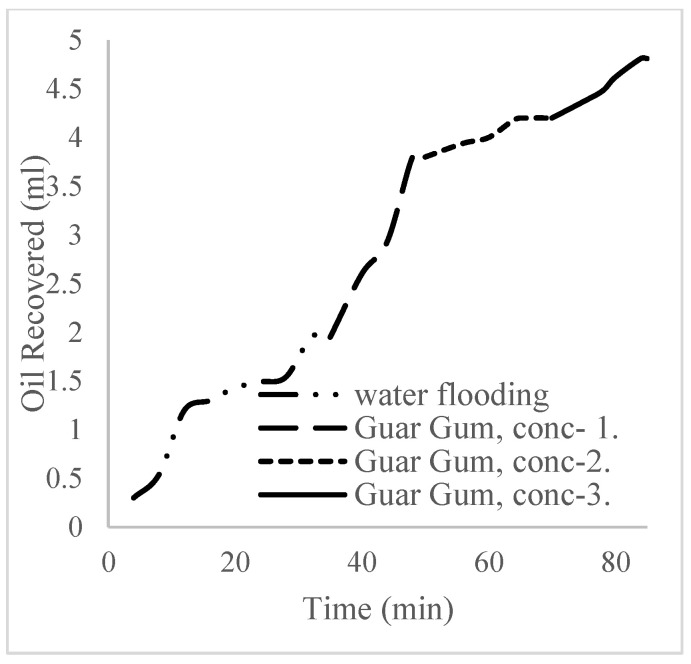
Oil recovery for 2 wt% of the polymer.

**Figure 18:**
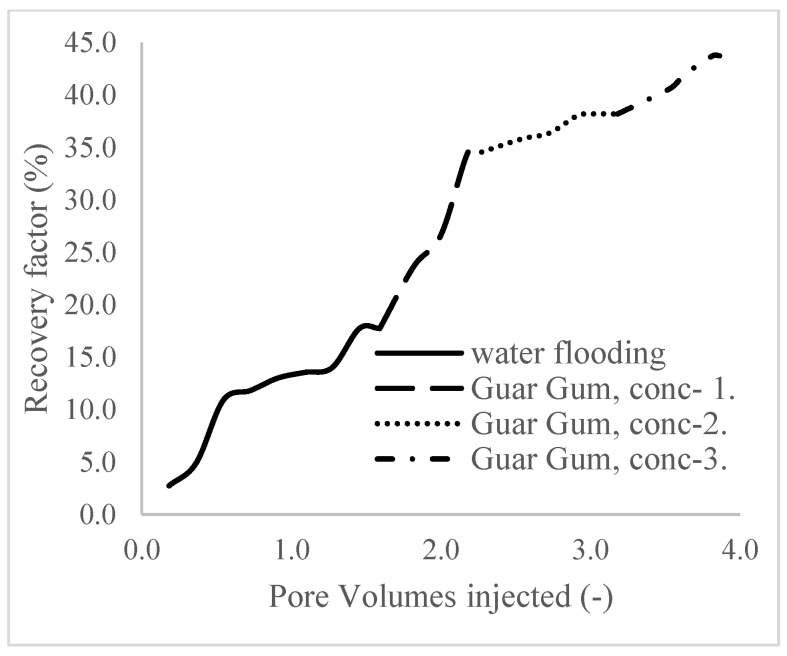
Oil recovery with respect to the pore volumes injected for case 1.

The plots in Figure 14 show the trend of oil recovery via water to polymer injection at different concentrations. A total of 1.6 pore volumes of water were injected at 3 mL/min. A total of 1.95 mL of oil was recovered during this process giving an oil recovery factor of 17.7% at the end of a water breakthrough time of 4 min (Figure 14). The first concentration of Guar gum (1% wt.) was introduced to flood 2.3 volumes of the pores and increased the oil recovery to 3.8 mL (Figure 15) giving an oil recovery of 34.5%. The second and third weight percentages of Guar gum were introduced to flood 3.2 and 3.9 pores of the core sample, respectively. The oil produced and recovery factors for the second polymer injections were 4.2 mL and 34.7% and for the third were 4.81 mL and 43.7% (Figure 16 and Figure 17). The incremental oil recoveries recorded for the water flooding for the polymer concentrations at 1 wt%, 1.5 wt%, and 2 wt% were 16.8%, 17%, and 26%, respectively and at a water breakthrough time of 50 min, 70 min, and 85 min. This suggests that increasing the polymer concentrations would ultimately increase the oil recovery due to the increase in viscosity of the injection phase. Figure 17 and Figure 18, respectively, describe the full trend of the oil recovery and oil recoveries per injected pore volume. Table 5 shows the summary of the cumulative oil recovery via each flooding procedure. 

### 3.2. Case 2

**Figure 19:**
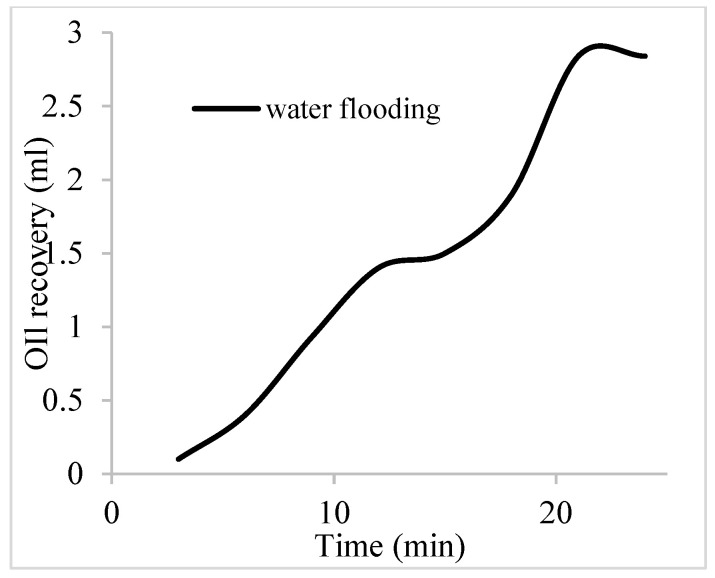
Oil recovery via water flooding.

The water flooding results from core C show an early oil production after 3 min and oil recovery with a factor of 26.8% and oil production of 2.4 mL at 1.1 per volume injected (Figure 19). At the onset of water production (24 min), a polymeric surfactant mixture of 5 wt% and 1 wt% of surfactant and polymer was injected. These processes were repeated for the polymeric surfactant mixtures of 5 wt% and 1.5 wt% (Conc. 2) and 5 wt% and 2 wt% (Conc. 3). These processes were commenced at the onset of water production (36, 51, and 63 min) or plateau oil production for each concentration. The results for the oil recovery factors and production are shown in Table 6.

**Figure 20:**
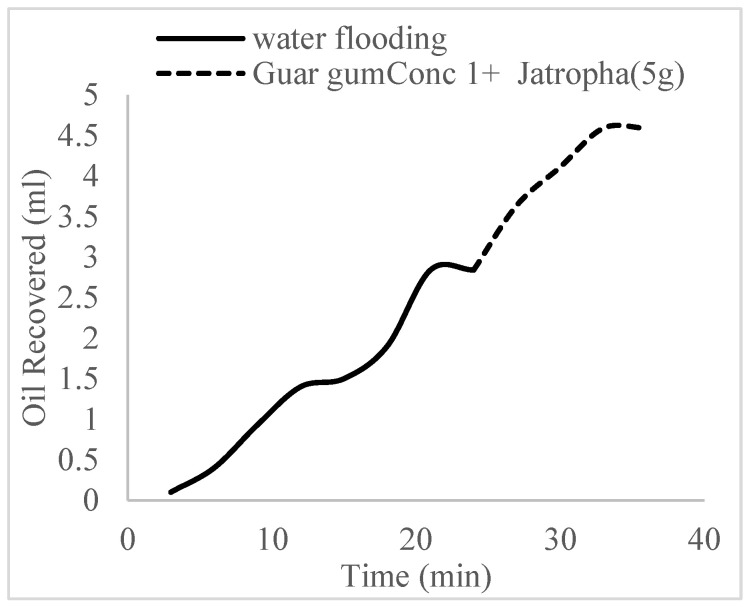
Oil recovery for PS (1 wt.% and 5 wt.%).

**Figure 21:**
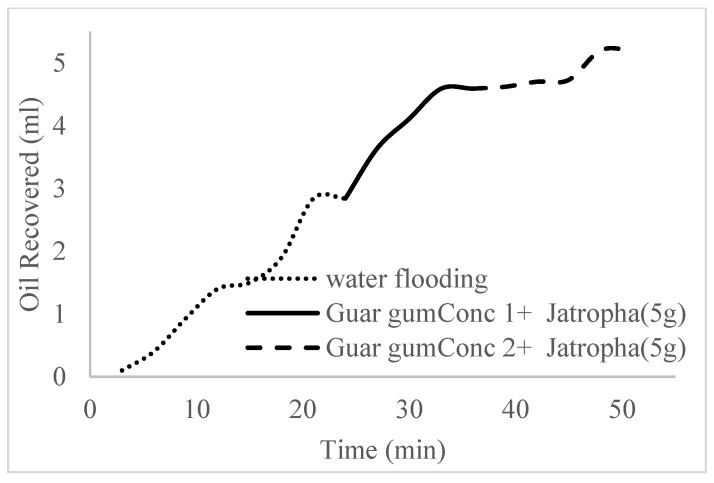
Oil recovery for PS (1.5 wt.% and 5 wt.%).

**Figure 22:**
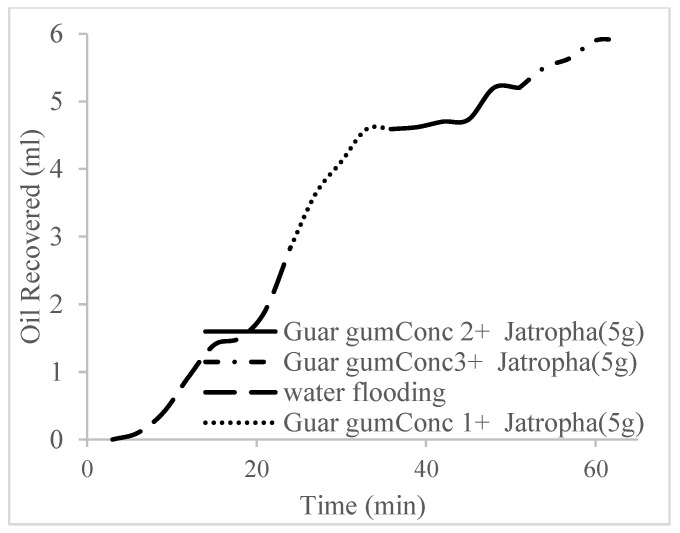
Oil recovery for PS (2 wt.% and 5 wt.%).

**Figure 23:**
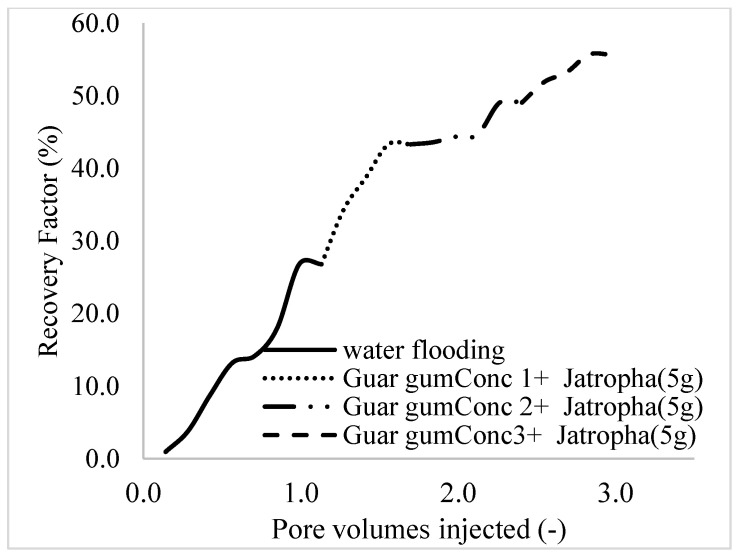
Oil recovery with respect to the pore volumes injected for case 2.

The trends of oil recovery for polymeric surfactants Conc. 1, Conc. 2, and Conc. 3 are described in Figure 20, Figure 21, Figure 22 and Figure 23. The incremental oil recovery factor recorded for water flooding for Conc. 1, Conc. 2, and Conc. 3 were 16.5%, 22.3%, and 28.9%, respectively.

### 3.3. Case 3

**Figure 24:**
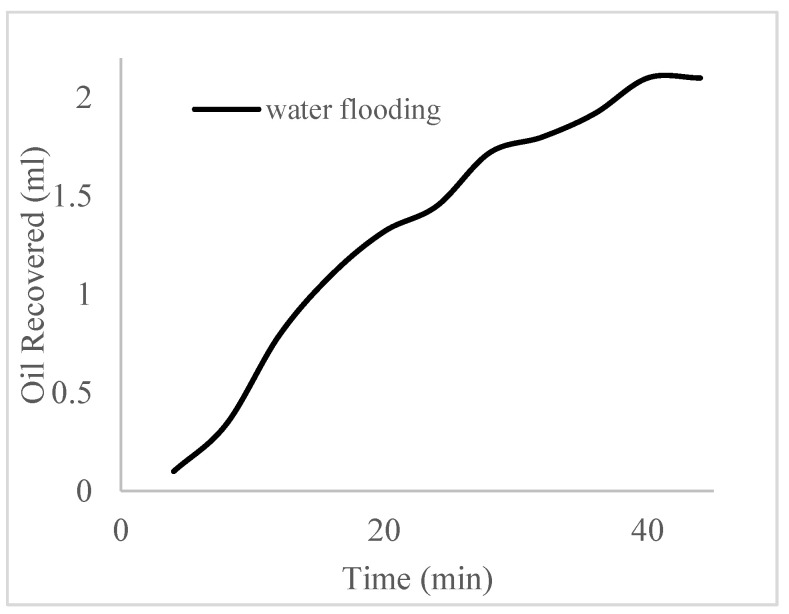
Oil recovery via water flooding.

**Figure 25:**
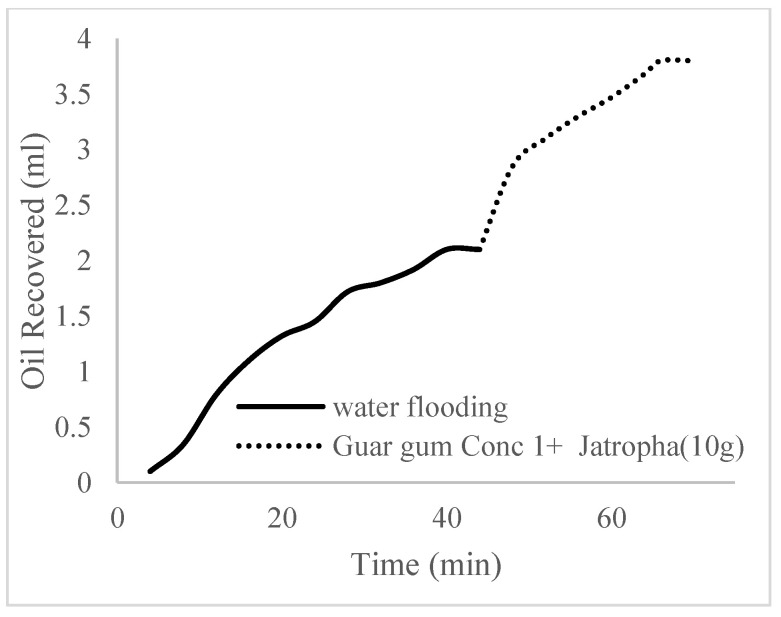
Oil recovery for PS (1 wt.% and 10 wt.%).

**Figure 26:**
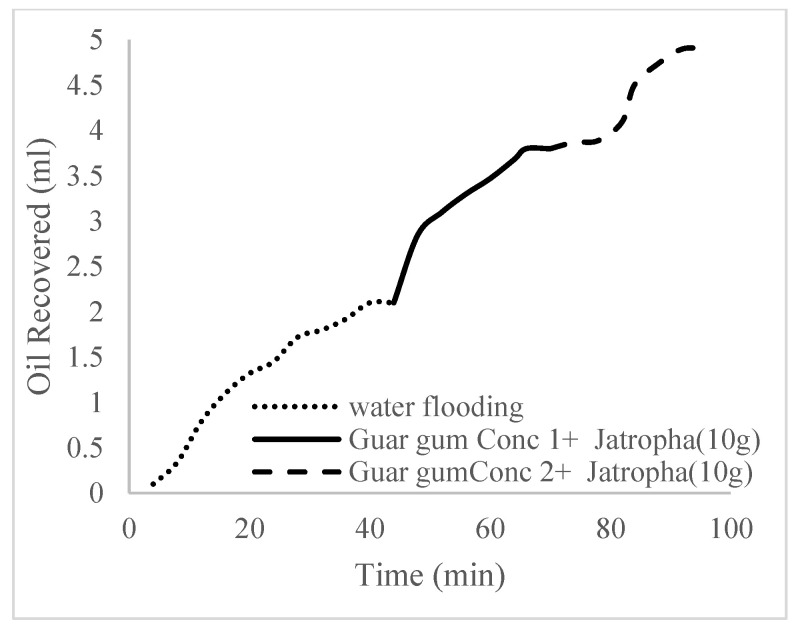
Oil recovery for PS (1.5 wt.% and 10 wt.%).

**Figure 27:**
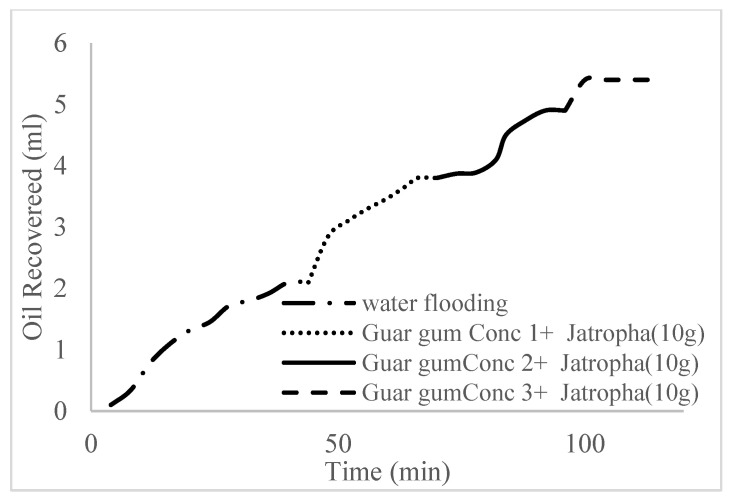
Oil recovery for PS (2 wt.% and 10 wt.%).

**Figure 28:**
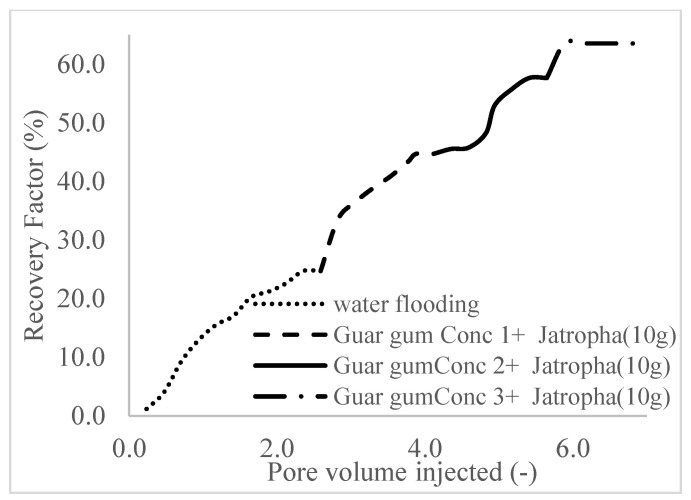
Oil recovery with respect to the pore volumes injected for case 3.

This case scenario involved the injection of the PS at a surfactant concentration of 10 wt%. The water flooding results conducted on sample K recovered 2.1 mL of oil at a factor of 24.7% after 44 min. The case scenario was commenced once oil production for a phase dropped or the water production increased. Three concentrations of polymeric surfactants of 10 wt% and 1 wt% (Conc. 1), 10 wt% and 1.5 wt% (Conc. 2), and 10 wt% and 2 wt% (Conc. 3) were introduced sequentially at the onset of the depletion of each phase. The total pore volumes injected, recovery factors, incremental recovery factors, and oil produced are summarized in Table 7. A longer water breakthrough time (water coning) of 70 min, 92 min, and 116 min was recorded as the concentrations of the PS increased. The oil recovery trends for the water flooding and polymeric surfactant injections of Conc. 1, Conc. 2, and Conc. 3 are described in Figure 24, Figure 25, Figure 26 and Figure 27 while Figure 28 shows the oil recovery factor trend against the pore volumes injected.

**Figure 29:**
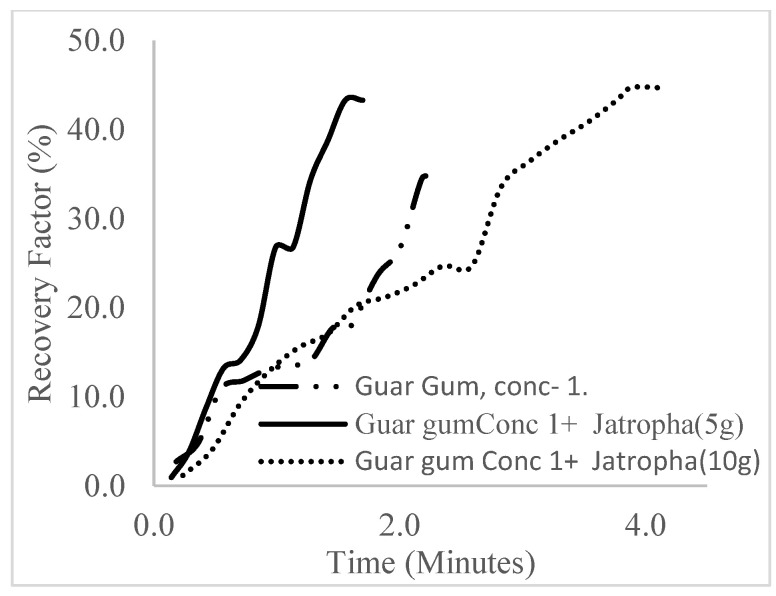
Oil recovery for polymeric surfactant Case 1.

**Figure 30:**
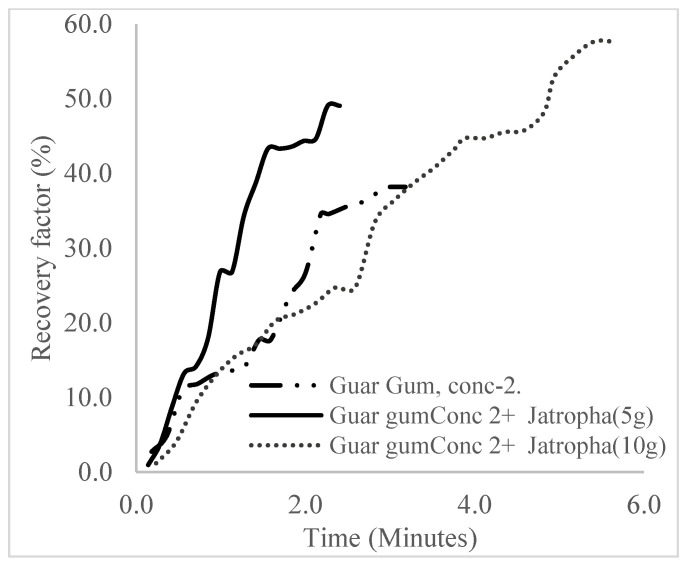
Oil recovery for polymeric surfactant Case 2.

**Figure 31:**
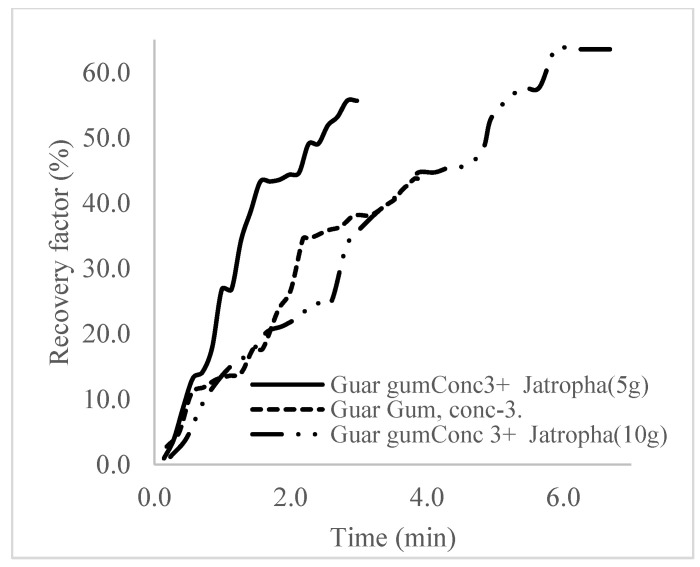
Oil recovery for polymeric surfactant Conc. 3.

**Figure 32:**
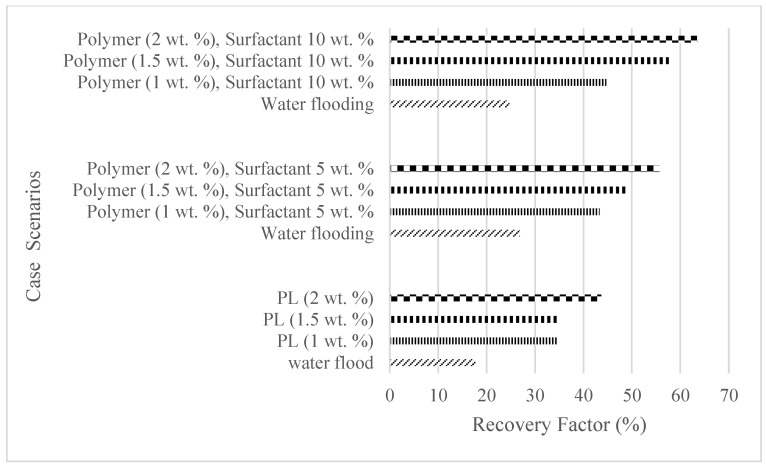
Summary of the oil recoveries from the case studies.

The plots in Figure 29, Figure 30 and Figure 31 compare the oil recovery trends between the three polymer concentrations and the surfactant concentrations. The plots show that increasing polymer concentrations increases the oil recoveries, and the incremental addition of weight percentages of surfactant during the flooding process further increases the oil recovery factors. The bar chart in Figure 32 shows the oil recovery for all the case scenarios considered.

## 4. Conclusions and Recommendation

The study has shown that Guar gum can increase the oil recovery by up to 26% at 2.0 wt% in comparison to secondary water flooding. The surfactants derived from Jatropha seeds, which are environmentally and economically friendly, can be mixed at different concentrations with polymers to form polymeric surfactants. An increase in the concentration of these mixtures will increase the ultimate oil recovery and as recorded from polymeric surfactant concentrations of (1.5 wt% and 10 wt%) and (2.0 wt% and 10 wt%), oil recoveries of 57.6% and 63.5% were recorded, respectively, resulting in an incremental recovery of 32.9% and 38.8% compared to water flooding. Despite the different experimentations offered by [36,39,40,41,42,43] to obtain oil recoveries from different PS concentrations, the results obtained in this article uniquely follow the similar trends of substantial incremental oil recoveries during PS injection compared to water and polymer injections. The in situ interfacial tension should be investigated to confirm the optimum surfactant concentration, and since a highly soapy effect was noticed with increased surfactant concentration, a flooding scenario adopting water alternating with a surfactant is recommended to reduce the soapy effects of the surfactants. Also, critical polymer concentrations need to be determined to counter the reduction in permeability values in reservoirs due to the high polymer adsorption rates. To carry out field assessments of the polymeric surfactants, the proper analysis of core flooding should be conducted using sensitivity analysis or the design of experiments as described by [46] based on different types of biopolymers and biosurfactants. This will assist in selecting the best polymeric surfactant to use. The field deployment can also be enhanced based on the results obtained from a reservoir simulation. This is of importance as the operational parameters such as production and injection rates, well trajectories, and well placements can be fully accounted for and optimized to further increase the oil recovery [47]. Other cheaper options for increasing the oil recovery in medium to heavy oil reservoirs include low-salinity water flooding [48]. Future research can also extend to the implementation of combined chemical-enhanced oil recovery techniques like alkaline surfactant polymer flooding.

## Figures and Tables

**Table 1:** Polymer concentrations.

Polymer Concentrations (g/cc)	2.0	1.5	1.0	0.5

**Table 2:** Characteristics of all the cores.

Sample	Length (cm)	Diameter (cm)	Pore Vol. (mL)	Bulk Vol. (mL)	Poro.	Perm.	Initial Oil Vol.	S_o_	S_w_
Core C	4.7	3.5	10.6	45.2	0.23	285	23.13	0.51	0.49
Core K	2.2	3.5	9	21.1	0.42	290	11.69	0.55	0.45
Core Z	5	3.5	11.5	48.1	0.2	158	26.78	0.56	0.44

**Table 3:** Physicochemical properties of the sulfonated surfactant.

Property	Value
Acid value (mg KOH/g of oil)	6.04
Color	Golden-yellow
Odor	Unpleasant
pH	6.34
Saponification value (mg KOH/g of oil)	198.69
Specific gravity	0.92
Viscosity at room temperature (cp)	36

**Table 4:** Fatty acid composition of the sulfonated surfactant.

Saturated	Fatty Acid	Percentage (%)
C16:0	Palmitic acid	13.75
C18:0	Stearic acid	6.68
**Unsaturated**		
C18:1	Oleic acid	44.16
C18:2	Linoleic acid	34.75
C16:1	Palmitoleic acid	0.65

**Table 5:** Summary of oil recovery for case scenario 1.

	Oil Recovery (%)	Incremental Oil Recovery (%)	Pore Vol. Injected (mL)	Oil Recovered (mL)	Residual Vol. (mL)
Water flooding	17.7	0	1.6	1.95	24.83
Polymer (1 wt%)	34.5	16.8	2.3	3.8	22.98
Polymer (1.5 wt%)	34.7	17	3.2	4.2	22.58
Polymer (2 wt%)	43.7	26	3.9	4.81	21.97

**Table 6:** Summary of the oil recovery for case scenario 2.

	Oil Recovery (%)	Incremental Oil Recovery (%)	Pore Volume Injected	Oil Recovered (mL)	Residual Volume
Water flooding	26.8	0	1.1	2.84	20.29
Polymer (1 wt%), Surfactant (5 wt%)	43.3	16.5	1.7	4.59	18.54
Polymer (1.5 wt%), Surfactant (5 wt%)	49.1	22.3	2.4	5.2	17.93
Polymer (2 wt%), Surfactant (5 wt%)	55.7	28.9	3.0	5.9	17.23

**Table 7:** Summary of the oil recovery for case scenario 3.

	Oil Recovery (%)	Incremental Oil Recovery (%)	Pore Vol. Inj.	Oil Recovery (mL)	Residual Volume
Water flooding	24.7	0	2.6	2.1	9.59
Polymer (1 wt%), Surfactant (10 wt%)	44.7	20	4.1	3.8	7.89
Polymer (1.5 wt%), Surfactant (10 wt%)	57.6	32.9	5.6	4.9	6.79
Polymer (2 wt%), Surfactant (10 wt%)	63.5	38.8	6.8	5.4	6.29

## Data Availability

The original contributions presented in the study are included in the article, further inquiries can be directed to the corresponding author.

## References

[1] Khojastehmehr M., Madani M., Daryasafar A. (2019). Screening of enhanced oil recovery techniques for Iranian oil reservoirs using TOPSIS algorithm. Energy Rep..

[2] Campbell D.A. (1981). Enhanced Oil-recovery and Its Environmental and Economic Implications in the United States. Environ. Conserv..

[3] Kovscek A.R. (2012). Emerging challenges and potential futures for thermally enhanced oil recovery. J. Pet. Sci. Eng..

[4] Hirasaki G.J. (2011). Recent Advances in Surfactant EOR. SPE J..

[5] Hirasaki G., Zhang D.L. (2004). Surface Chemistry of Oil Recovery from Fractured, Oil-Wet, Carbonate Formations. SPE J..

[6] Samanta A., Ojha K., Sarkar A., Mandal A. (2011). Surfactant and Surfactant-Polymer Flooding for Enhanced Oil Recovery. Adv. Pet. Explor. Dev..

[7] Mokheimer E.M., Hamdy M., Abubakar Z., Shakeel M.R., Habib M.A., Mahmoud M. (2019). A comprehensive review of thermal enhanced oil recovery: Techniques evaluation. J. Energy Resour. Technol..

[8] Lazar I., Petrisor I.G., Yen T.F. (2007). Microbial Enhanced Oil Recovery (MEOR). Pet. Sci. Technol..

[9] Hadi S., Clarence A.M., Michael S.W., James M.T., Rafael V. (2014). Polymer Coated Nanoparticles for Enhanced Oil Recovery. J. Appl. Polym. Sci..

[10] Abraham V.D., Orodu O.D., Efeovbokhan V.E., Olabode O.A., Ojo T.I. (2020). The Influence of Surfactant Concentration and Surfactant Type on the Interfacial Tension of Heavy Crude Oil/Brine/Surfactant System. Pet. Coal..

[11] Olabode O.A. (2018). Production forecast for Niger delta oil rim synthetic reservoirs. Data Brief.

[12] Olabode O., Ojo T., Oguntade T., Oduwole D. (2020). Recovery potential of biopolymer (B-P) formulation from Solanum tuberosum (waste) starch for enhancing recovery from oil reservoirs. Energy Rep..

[13] Tabary R., Bazin B., Douarche F., Moreau P., Oukhemanou-Destremaut F. Surfactant Flooding in Challenging Conditions: Towards Hard Brines and High Temperatures. Proceedings of the SPE Middle East Oil and Gas Show and Conference.

[14] Arash R., Milad S., Ali N., Abdolnabi H., Shahab A. (2013). Core flooding tests to investigate the effects of IFT reduction and wettability alteration on oil recovery during MEOR process in an Iranian oil reservoir. Appl. Microb. Cell Physiol..

[15] Liu X. (2021). Experimental evaluation of polymer performance for tertiary oil recovery in oil field. IOP Conf. Ser. Earth Environ. Sci..

[16] Olajire A.A. (2014). Review of ASP EOR (alkaline surfactant polymer enhanced oil recovery) technology in the petroleum industry: Prospects and challenges. Energy.

[17] John M.F., Olabode O.A., Egeonu G.I., Ojo T.I. (2017). Enhanced Oil Recovery of Medium Crude Oil (31Api) Using Nanoparticles and Polymer. Int. J. Appl. Eng. Res..

[18] Temiouwa O., Oluwasanmi O., Ifeanyi S., Tomiwa O. Nano Augumented Biosurfactant Formulation for Oil Recovery in Medium Oil Reservoirs. Proceedings of the SPE Nigeria Annual International Conference and Exhibition.

[19] Zhang J., Misra R.D.K. (2007). Magnetic drug-targeting carrier encapsulated with thermosensitive smart polymer: Core–shell nanoparticle carrier and drug release response. Acta Biomater..

[20] Sidiq H., Abdulsalam V., Nabaz Z. (2019). Reservoir simulation study of enhanced oil recovery by sequential polymer flooding method. Adv. Geo-Energy Res..

[21] Olabode O., Akinsanya O., Daramola O., Sowunmi A., Osakwe C., Benjamin S., Samuel I. (2023). Effect of Salt Concentration on Oil Recovery during Polymer Flooding: Simulation Studies on Xanthan Gum and Gum Arabic. Polymers.

[22] Skauge T., Hetland S., Spildo K., Skauge A. Nano-sized particles for EOR. Proceedings of the SPE Improved Oil Recovery Symposium.

[23] Sensoy T., Chenevert M.E., Sharma M.M. Minimizing water invasion in shale using nanoparticles. Proceedings of the SPE Annual Technical Conference and Exhibition.

[24] Alvani A., Jouyban A., Shayanfar A. (2019). The effect of surfactant and polymer on solution stability and solubility of tadalafil-methylparaben cocrystal. J. Mol. Liq..

[25] Gogoi S.B. (2011). Adsorption–Desorption of Surfactant for Enhanced Oil Recovery. Transp. Porous Media.

[26] Madani M. (2019). Fundamental investigation of an environmentally friendly surfactant agent for chemical enhanced oil recovery. Fuel.

[27] Elraies K.A., Tan I.M., Fathaddin M.T., Abo-Jabal A. (2011). Development of a New Polymeric Surfactant for Chemical Enhanced Oil Recovery. Pet. Sci. Technol..

[28] Saxena N. (2019). Bio-based surfactant for enhanced oil recovery: Interfacial properties, emulsification and rock-fluid interactions. J. Pet. Sci. Eng..

[29] Pal N. (2019). Phase behaviour and characterization of micro emulsion stabilized by a novel synthesized surfactant: Implications for enhanced oil recovery. Fuel.

[30] Ayoub M. (2021). A Comprehensive Review on Oil Extraction and Biodiesel Production Technologies. Sustainability.

[31] Faria D. (2018). Extraction of radish seed oil (*Raphanus sativus* L.) and evaluation of its potential in biodiesel production. AIMS Energy.

[32] Muentes S.A.G., Ávila M.G.G., Vázquez B.L.L., del Campo Laffita A.E.S. (2017). The production of biodiesel from Jatropha curca and its social impact. Int. Res. J. Eng. IT Sci. Res..

[33] Castañeda I., Bojacá V., Ramos Borda G.E., Navarro S., Marìa A., Acevedo Pabon P.A. (2017). Study of the eco-efficiency of biodiesel production from the fruit of the *Jatropha curcas* plant. Chem. Eng..

[34] Huang J., Jiang P., Wen Y., Deng J., He J. (2016). Soy-castor oil-based polyurethanes with octaphenylsilsesquioxanetetraol double-decker silsesquioxane in the main chains. RSC Adv..

[35] Awang M., Diyanti I. (2010). The Production of Surfactant by Direct Reaction of Pyrolysis Oil for Enhanced Oil Recovery. Energy Sources Part A Recovery Util. Environ. Eff..

[36] Kumar S. (2016). Synthesis and evaluation of physicochemical properties of anionic polymeric surfactant derived from Jatropha oil for application in enhanced oil recovery. J. Ind. Eng. Chem..

[37] Fei D., Guo J., Xiong R., Zhang X., Kang C., Kiyingi W. (2023). Preparation and Performance Evaluation of Amphiphilic Polymers for Enhanced Heavy Oil Recovery. Polymers.

[38] Lee E.M., Koopal L.K. (1996). Adsorption of Cationic and Anionic Surfactants on Metal Oxide Surfaces: Surface Charge Adjustment and Competition Effects. J. Colloid Interface Sci..

[39] Guo J., Wang F., Zhao Y., Wang P., Wang T., Yang J., Yang B., Ma L. (2024). A Laboratory Experimental Study on Enhancing the Oil Recovery Mechanisms of Polymeric Surfactants. Molecules.

[40] Wibowo A.D.K., Megawati R., Setyaningrum V.K., Putri E.W., Handayani A.S., Solikhah M.D., Chafidz A. (2023). Investigating potential application of bio-based polymeric surfactant using methyl ester from palm oil for chemical enhanced oil recovery (CEOR). Commun. Sci. Technol..

[41] Wibowo A.D.K., Yoshi L.A., Handayani A.S., Joelianingsih. (2021). Synthesis of polymeric surfactant from palm oil methyl ester for enhanced oil recovery application. Colloid Polym. Sci..

[42] Li B., Liu Z., Fei C., Lv J., Chen X., Li Y. Polymeric Surfactant for Enhanced Oil Recovery-Microvisual, Core-Flood Experiments and Field Application. Proceedings of the SPE EOR Conference at Oil and Gas West Asia.

[43] Babu K., Pal N., Saxena V.K., Mandal A. (2016). Synthesis and characterization of a new polymeric surfactant for chemical enhanced oil recovery. Korean J. Chem. Eng..

[44] Olabode O.A. (2020). Effect of water and gas injection schemes on synthetic oil rim models. J. Pet. Explor. Prod. Technol..

[45] Sharma T., Suresh Kumar G., Sangwai J.S. (2014). Enhanced oil recovery using oil-in-water (o/w) emulsion stabilized by nanoparticle, surfactant, and polymer in the presence of NaCl. Geosyst. Eng..

[46] Massarweh O., Abushaikha A.S. (2020). The use of surfactants in enhanced oil recovery: A review of recent advances. Energy Rep..

[47] Olabode O.A., Isehunwa S., Orodu O., Rotimi T., Mamudu A. (2018). Effect of foam and WAG (water alternating gas) injection on performance of thin oil rim reservoirs. J. Pet. Sci. Eng..

[48] Yan L. (2020). Impact of water salinity differential on a crude oil droplet constrained in a capillary: Pore-scale mechanisms. Fuel.

